# Effect of Optimization of TiO_2_ Electron Transport Layer on Performance of Perovskite Solar Cells with Rough FTO Substrates

**DOI:** 10.3390/ma13102272

**Published:** 2020-05-15

**Authors:** Junqi Wang, Xiaoping Zou, Jialin Zhu, Jin Cheng, Dan Chen, Xiao Bai, Yujun Yao, Chuangchuang Chang, Xing Yu, Baoyu Liu, Zixiao Zhou, Guangdong Li

**Affiliations:** 1 Beijing Advanced Innovation Center for Materials Genome Engineering, Research Center for Sensor Technology, Beijing Key Laboratory for Sensor, MOE Key Laboratory for Modern Measurement and Control Technology, School of Automation, Beijing Information Science and Technology University, Jianxiangqiao Campus, Beijing 100101, China; 13126706081@163.com (J.W.); chengjin@bistu.edu.cn (J.C.); baixiao_edu@163.com (X.B.); yyj10zy@gmail.com (Y.Y.); changcc037@gmail.com (C.C.); nimingyx1@163.com (X.Y.); liubaoyu0214@163.com (B.L.); zzxfpp111@163.com (Z.Z.); LGD1511455720@163.com (G.L.); 2 State Key Laboratory on Integrated Optoelectronics, Institute of Semiconductors, Chinese Academy of Sciences, Beijing 100083, China; chendan1988@semi.ac.cn; 3 Center of Materials Science and Optoelectronics Engineering, University of Chinese Academy of Sciences, Beijing 100049, China

**Keywords:** perovskite solar cell, TiO_2_ electron transport layer, FTO substrate

## Abstract

The film quality of the electron transport layer (ETL) plays an important role in improving the performance of perovskite solar cells (PSCs). In order to reduce the effect of rough fluorine-doped SnO_2_ (FTO)substrate on the film quality of the TiO_2_ ETL, multiple cycles of spin-coating were employed to realize optimized TiO_2_ film and improve the performance of PSCs with rough FTO. The results show that TiO_2_ ETL was optimized most effectively using two spin-coating cycles, obtaining the best performance of PSCs with rough FTO. The carbon electrode-based PSCs were then demonstrated. Our work discusses the feasibility of low-quality rough FTO for the fabrication of PSCs and photodetectors to reduce costs.

## 1. Introduction

Solar cells are rapidly improving [1,2]. Recently, perovskite solar cells (PSCs) have drawn increasing attention due to their high power conversion efficiency (PCE), low cost, and facile preparation process [3,4,5,6,7,8]. A conventional planar heterojunction PSC is composed of a counter electrode (cathode), a hole transport layer, a perovskite layer, an electron transport layer (ETL), and a conductive glass substrate [9,10,11]. The ETL plays an important role in extracting electrons from the perovskite layer. It can prevent contact between the fluorine-doped SnO_2_ (FTO) and perovskite layer to avoid the recombination of electrons and holes, and reduce energy loss at the interface [11,12,13,14,15,16,17].

There are various preparation processes for the electron transport layer, including pyrolytic spraying [18], atomic layer deposition [19], thermal oxidation [20], electrochemical deposition, and liquid-phase spin-coating. The most widely used and simplest process is the liquid-phase spin-coating method [21,22,23].

It is essential to form appropriate ETL thickness to extract electrons and block holes effectively. Thick ETLs may cause high series resistance, which could deteriorate the performance of PSCs. Thin ETLs may not passivate all defects, and current leakage may occur. Any pinholes in ETLs can lead to shunt pathways and direct contact between the perovskite layer and conductive glass, causing high leakage current and severe carrier recombination at the interface [24]. It is reported that the loss of energy in PSCs corresponds to the recombination of electrons and holes in the carrier transport from perovskite to electrode [25,26]. Due to these restrictions, a single ETL cannot suppress the leakage current. It is generally known that charge recombination is highly responsible for reducing the energy conversion efficiency of PSCs. Many researchers have focused on the ETL of PSCs [16,17,27]. Some investigators have tried to add a new layer into the ETL to form a bilayer, which has been demonstrated as an effective way to modify the interfacial behavior and photovoltaic performance of PSCs. Chen et al. optimized the surface of ZnO with 3-aminopropanioc acid to improve the performance of solar cells [28]. Guo et al. added bathocuproine film as a hole-blocking layer between PCBM and Al to block holes in PSCs with an inverted structure [29]. They focus on the interface between the ETL and perovskite of the PSCs, giving little attention to the interface between ETLs and electrodes. Fang et al. deposited an MgO layer on the anode surface as a hole-blocking layer to avoid the recombination of electrons and holes [15].

In order to further improve the performance of the perovskite solar cell prepared with the liquid-phase spin-coating process, it is necessary to optimize the preparation process of the ETL to make the film uniform, non-porous, and more conductive [30].

However, the film is spin-coated on a rough FTO substrate, leading to uneven thickness, pinholes, and incomplete surface coverage of the TiO_2_ layer. This will result in contact between the perovskite light-absorption layer and the FTO substrate, resulting in the recombination of electrons and holes at the interface [31].

In our work, the problem of rough FTO substrate is solved by spin-coating TiO_2_ for different cycles on the FTO substrate. The TiO_2_ ETL was optimized to form a thin film without holes and cracks. The solar cell device prepared with the optimized TiO_2_ layer displayed the best electrical performance, with small series resistance and large shunt resistance obtained by SEM, XRD, and current–voltage (J–V) curve measurements. Finally, the carbon-electrode-based PSCs are demonstrated. Our work expands the feasibility of using low-quality FTO for the fabrication of PSCs and photodetectors to reduce costs.

## 2. Experiments

### 2.1. Materials

Acidic titanium dioxide solution (HH-TiO*x*), *N*,*N*-dimethylformamide (DMF), dimethyl sulfoxide (DMSO), and isopropyl alcohol (IPA) were purchased from Shanghai MaterWin New Materials Corp. (Shanghai, China). Methylammonium iodide (MAI), PbI_2_, fluorine-doped SnO_2_ (FTO) substrates, and spiro-MeOTAD solution were purchased from Xi’an Polymer Light Technology Corp. (Xi’an, China).

### 2.2. Device Fabrication

**Figure 1:**
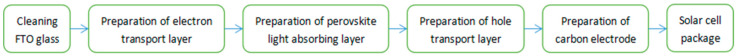
Flow schematic of device fabrication.

A flow schematic of the device fabrication is shown in Figure 1. The detailed experimental process is described as follows.

#### 2.2.1. Cleaning FTO Glass

Surface treatment of the FTO substrate is essential before the deposition of any film to ensure complete coverage, and can severely affect the film deposition and properties. Malviya et al. demonstrated that rigorous cleaning processes yielded a clean FTO surface, and found that rigorous cleaning of the substrates prior to hematite deposition was crucial for achieving highly reproducible results [32].

In this work, we first cut the FTO rigid substrate into squares with an area of 1.5 cm × 1.5 cm. We placed the cut substrates with the conductive surface facing up in a single layer in a cleaned Petri dish, added an appropriate amount of deionized water and detergent to the Petri dish, then put them in an ultrasonic vibration cleaner for 20 min. We rinsed the substrate surfaces with a large amount of deionized water to remove substances that were soluble in the detergent. Following this, we added an appropriate amount of absolute ethanol to the Petri dish and ultrasonically cleaned the substrates for 20 min to remove various impurities on the surfaces that were easily soluble in absolute ethanol. We then rinsed the substrate surfaces with a large amount of deionized water to ensure no ethanol remained. Next, we added an appropriate amount of mixed solution of isopropanol, acetone, and deionized water with a volume ratio of 1:1:1 to the Petri dish, and ultrasonically washed the substrates for 20 min to remove various impurities on the surface that were easily soluble in isopropanol and acetone. After this process, the surfaces of the conductive substrate were washed with a large amount of deionized water to ensure no residual isopropanol and acetone remained. The cleaned substrates were dried in a constant-temperature drying box for 90 min, then placed in a UV light washer for 15 min.

#### 2.2.2. Preparation of Electron Transport Layer

The dense TiO_2_ layer was spin-coated on an FTO substrate with an acidic TiO_2_ solution at a spin-coating rate of 2000 rpm for 60 s. The spin-coated FTO substrate was then annealed on a hot plate at 150 °C for 20 min. Finally, it was sintered in a muffle furnace at 500 °C for 30 min. The dense TiO_2_ layer was spin-coated 1~3 times and was sintered after each spin-coating.

#### 2.2.3. Preparation of Perovskite Absorption Layer

The lead iodide (PbI_2_) precursor solution was a mixed solution of PbI_2_ dissolved in a mixed solution of DMF and DMSO, where the volume ratio of DMF to DMSO was 0.95:0.05 in the 600 mg/mL precursor solution. The methyl iodide (CH_3_NH_3_I/MAI) precursor solution was a solution of 70 mg/mL MAI dissolved in anhydrous isopropanol. The PbI_2_ precursor solution was directly spin-coated on the dense layer at the rate of 1500 rpm for 30 s. When the rotation was stopped, the MAI precursor solution was uniformly drip-coated on the PbI_2_ film with a pipette, and then immediately spin-coated at the rate of 1500 rpm for 30 s. The spin-coated substrate was annealed on a hot-plate at 150 °C for 20 min.

#### 2.2.4. Preparation of Hole Transport Layer

A volume of 20 µL of 2,2′,7,7′-tetrakis(*N*,*N*-di-p-methoxyphenylamine)-9,9-spirobifluorene (spiro-MeOTAD) solution was spin-coated on the CH_3_NH_3_PbI_3_ perovskite layer at 3000 rpm for 30 s. A spiro-MeOTAD solution was prepared by dissolving 72.3 mg of spiro-MeOTAD in 1 mL of chlorobenzene, to which 28.8 µL of 4-tert-butylpyridine and 17.5 µL of lithiumbis (trifluoromethanesulfonyl) imide (Li-TFSI) solution (520 mg Li-TSFI in 1 mL acetonitrile, 99.8%) were added [33].

#### 2.2.5. Preparation of Carbon Film Counter Electrode

In this paper, a carbon/FTO composite counter electrode was used as the photoanode of the PSCs. We adopted the preparation process reported by Zhang et al. [34]. The detailed steps were as follows: An external flame was used to smoke the conductive surface of the cleaned FTO glass substrate. During the fumigation process, we continuously moved the FTO substrate back and forth to cover the carbon film uniformly. After 5 to 7 s, we removed the FTO substrate from the flame and preparation of carbon film counter electrode was completed.

#### 2.2.6. Solar Cell Package

The carbon film counter electrode was placed against the hole transport layer. The two substrates were compacted tightly to ensure they did not move, and clamped with a dovetail clip. A suitable mask was then added on the photoanode surface to ensure that the solar cell had an accurate and effective working area.

### 2.3. Characterization

An X-ray diffractometer (XRD) (Broker, D8 Focus, Dresden, Germany) was used to obtain XRD spectra from samples of TiO_2_ films deposited on FTO substrates. Field emission scanning electron microscope (FE-SEM) (SU8020, Hitachi, Tokyo, Japan) images were obtained for structure and morphology characterization of the TiO_2_ films, FTO substrate, and perovskite solar cell. The J–V curves were obtained under standard simulated air-mass (AM) 1.5 sunlight generated from a solar simulator (Oriel Sol3A, Newport, RI, USA). All characterizations of devices were performed in the ambient atmosphere at room temperature.

## 3. Results and Discussion

**Figure 2:**
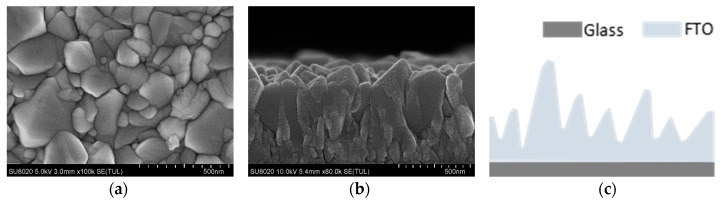
SEM images of top view (**a**), cross section (**b**), and schematic diagram (**c**) of the rough surface of the FTO substrate.

Top-view and cross section SEM images of the FTO substrate and a schematic diagram of the rough surface of the FTO are shown in Figure 2a–c, respectively. As illustrated in Figure 2, the FTO substrates we used had rough surfaces [31], which will cause cracks and holes in the TiO_2_ ETL spin-coated on the FTO substrate and may affect the performance of the solar cells. In order to reduce the effect of the rough FTO substrate on the TiO_2_ film, the TiO_2_ films were spin-coated for multiple cycles on a rough FTO substrate.

**Figure 3:**
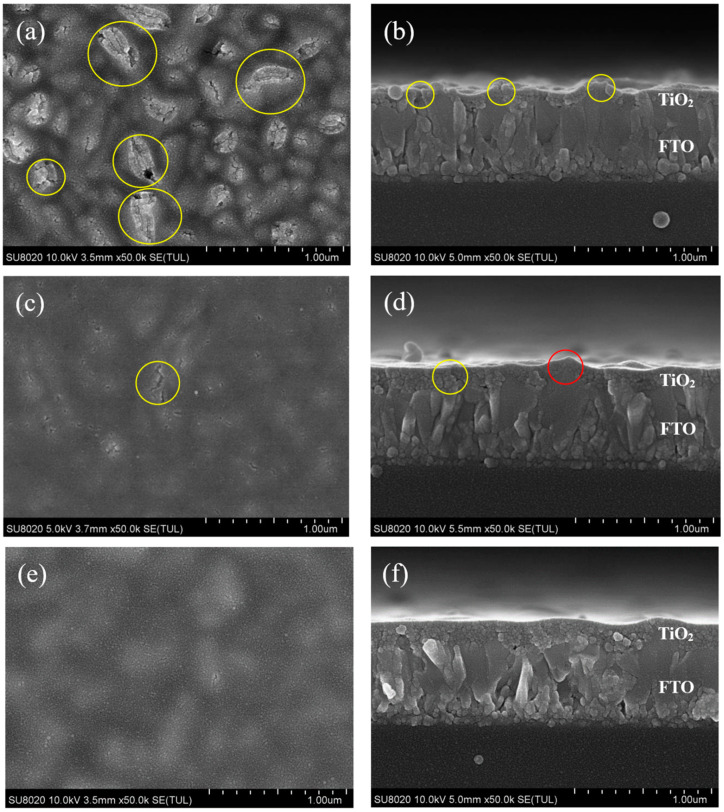
SEM images of TiO_2_ film with different spin-coating cycles: (**a**,**c**,**e**) Top view images of TiO_2_ for one, two, and three spin-coating cycles, respectively; (**b**,**d**,**f**) Cross-sectional view images of TiO_2_ for one, two, and three spin-coating cycles, respectively.

SEM images of TiO_2_ films with different numbers of spin-coating cycles are shown in Figure 3. Figure 3a,b respectively shows the top and cross-sectional views of TiO_2_ film with one spin-coating cycle. As shown in the figure, the film was undulating, and there were many cracks (as shown by the yellow circle in Figure 3a,b). The cracks are attributed to the rough surface of the FTO substrate. The TiO_2_ film generated large stress at the peaks of FTO, which could cause cracking during heating.

When TiO_2_ was spin-coated once, the thin film could not completely cover the peaks of FTO, as shown in Figure 3a,b. This will result in the FTO having direct contact with the perovskite layer, so the performance of solar cell devices may be poor.

Figure 3c,d are top-view images and cross-sectional images of two spin-coating cycles of TiO_2_, respectively. According to Figure 3c,d, the film was flatter than with one spin-coating cycle of TiO_2_, and there were few cracks in the film (as shown by the yellow circle in Figure 3c,d).

After spin-coating TiO_2_ twice, the FTO substrate was completely covered by the TiO_2_ film. Compared with one spin-coating cycle of TiO_2_ film, FTO peaks were basically not visible in the top view, which is also confirmed by the corresponding cross-sectional images shown in Figure 3c,d. The high FTO peak at the center of the cross-sectional image had also been covered by TiO_2_ (as shown by the red circle in Figure 3d). The TiO_2_ film could prevent contact between FTO and the perovskite layer, so it may improve the device performance.

Figure 3e,f are top-view and cross-sectional images of three spin-coating cycles of TiO_2_, respectively. As illustrated in Figure 3e,f, the film was thicker than with two spin-coating cycles of TiO_2_.

After spin-coating TiO_2_ three times, the film was very flat and smooth, with a thickness of about 200 nm, which contributed to the growth of perovskite film, as shown in Figure 3e,f. However, compared with two spin-coating cycles of TiO_2_ film, thicker TiO_2_ film made it less effective for electrons to be injected from the perovskite layer to the FTO substrate, which may deteriorate the performance of the device.

**Figure 4:**
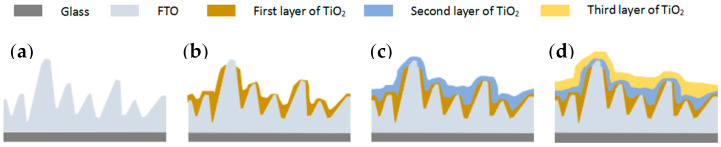
Schematic diagram of multiple layers of TiO_2_ on rough FTO substrate. (**a**): rough FTO; (**b**): 1 layer of TiO_2_ on the FTO; (**c**): 2 layer of TiO_2_ on the FTO; (**d**): 3 layer of TiO_2_ on the FTO.

The schematic diagram extracted from the cross-sectional SEM images in Figure 3 is shown in Figure 4.

**Figure 5:**
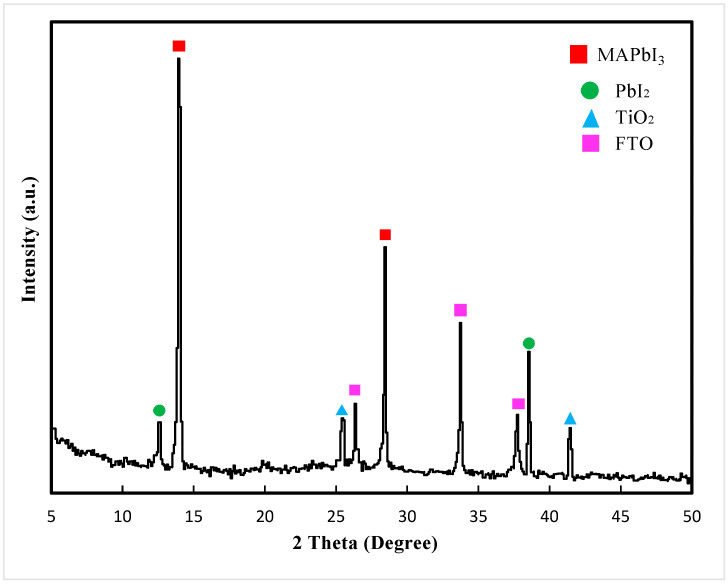
XRD pattern of perovskite light-absorbing layer film.

The XRD pattern of the perovskite light-absorbing layer film is shown in Figure 5. The characteristic diffraction peaks were located at 2θ = 14.19° and 28.50°, which corresponds to the (110) and (220) planes of the perovskite crystal planes [35,36], respectively. As shown in Figure 5, MAPbI_3_ had a tetragonal perovskite structure.

**Figure 6:**
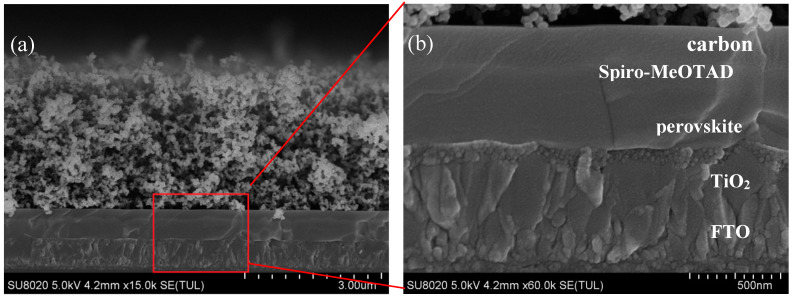
(**a**) Cross-sectional SEM images of perovskite solar cell with two cycles of spin-coating TiO_2_; (**b**) An enlargement of the red frame in (**a**).

**Figure 7:**
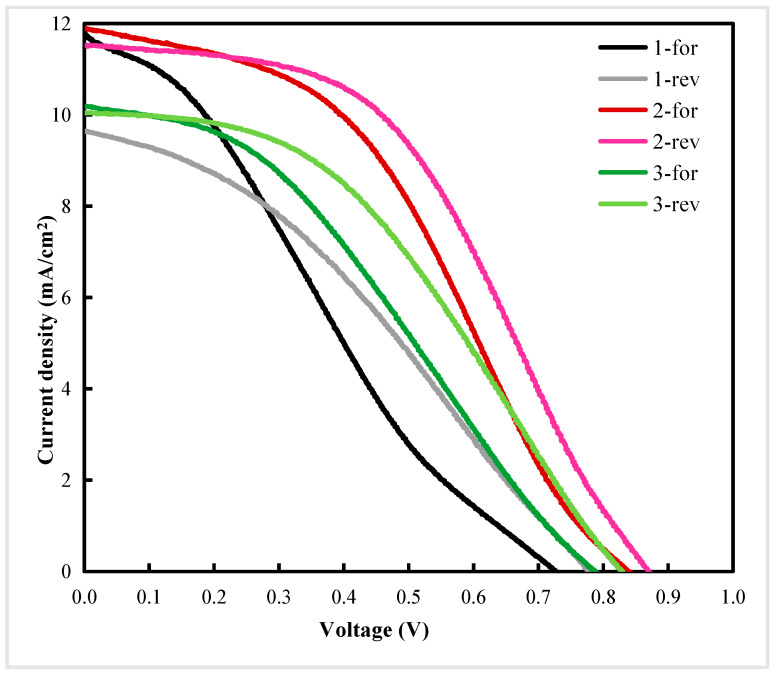
Current–voltage (J–V) curves of perovskite solar cells with different spin-coating cycles.

Cross-sectional SEM images of a perovskite solar cell with two cycles of spin-coated TiO_2_ are shown in Figure 6, where Figure 6b displays the magnified diagram of the marked area in the red rectangle in Figure 6a. The dense TiO_2_ layer was prepared by two spin-coating cycles. As shown in Figure 7, the TiO_2_ layer was dense and uniform; the grains of the perovskite layer were complete, with a thickness of about 400 nm; the thickness of the spiro-MeOTAD layer was about 250 nm; the top layer was a spongy-like carbon film with a thickness of about 3 μm.

The J–V curves of PSCs with different spin-coating cycles are shown in Figure 7. As shown in Table 1, the performance parameters corresponded to the J–V curves in Figure 7. The series resistance and shunt resistance were calculated from the J–V curves. In this paper, -for and -rev represent forward-scanning and reverse-scanning, respectively. In Figure 7, 1-, 2-, and 3-represent the number of TiO_2_ spin-coating cycles. It can be seen from Table 1 that the performance of PSCs prepared with spin-coating for two cycles was better than others.

As shown in Table 1 and Figure 3a, compared with spin-coating for two cycles, when TiO_2_ films were fabricated using one spin-coating cycle, the films could not completely cover the peaks of FTO, and had pinholes. Therefore, the films may not be capable of passivating the defects efficiently. Any pinholes in the TiO_2_ film could lead to shunt pathways and direct contact between the CH_3_NH_3_PbI_3_ light-absorption layer and FTO, resulting in high leakage current and serious charge carrier recombination at the interface [18]. A device with an excessively thin TiO_2_ film could not completely cover the FTO, and would experience serious charge carrier recombination, contributing to low shunt resistance and leading to a low open-circuit voltage (V_oc_). As such, the performance of PSCs prepared with spin-coating for two cycles is better than spin-coating for one cycle.

When the number of spin-coating cycles was increased to three cycles, the thickness of TiO_2_ increased, as shown in Figure 3f. This led to poor transmittance and weaker light absorption of the perovskite film. When the thickness of TiO_2_ was increased, the distance became too large to transfer electrons from the perovskite film to the FTO. Therefore, the electrons run a longer distance. Furthermore, the excessively thick ETL had a higher series resistance, which contributed to low short-circuit photocurrent density (J_sc_). Thus, the performance of PSCs prepared with spin-coating for two cycles was better than spin-coating for three cycles.

## 4. Conclusions

Using multiple-cycle spin-coated TiO_2_ films on rough FTO was demonstrated to reduce the effect of the rough FTO substrate and improve device performance for PSCs in this work.

It was found that the TiO_2_ ETL was optimized by using two spin-coating cycles. It could form a thin film without holes and cracks, avoiding contact between FTO and the perovskite layer to mitigate the recombination of electrons and holes and reduce energy loss at the interface. The solar cell device prepared with an optimized TiO_2_ layer had the best electrical performance, with small series resistance and large shunt resistance, so it could improve the performance of PSCs.

The influence of rough FTO on the TiO_2_ film can be reduced using this optimized method, meaning low-cost and low-quality FTO substrates can be used to fabricate solar cells or photodetectors.

However, there were still some points that need to be improved in our research; for example, an excessively high annealing temperature may degrade MAPbI_3_. An overly thick spiro-MeOTAD layer may also have some impact on the device, which was based on a carbon electrode in our work. Gold electrodes, the optimization of annealing temperature of MAPbI_3_, and spiro-MeOTAD layer thickness are currently under investigation.

## Figures and Tables

**Table 1:** Performance parameters of perovskite solar cells with different spin-coating cycles of TiO_2_.

Sample	PCE ^a^ (%)	V_oc_^b^ (V)	J_sc_^c^ (mA/cm^2^)	FF ^d^	R_s_^e^ (Ωcm^2^)	R_sh_^f^ (Ωcm^2^)	Average Thickness ^g^ (nm)	Standard Error of Thickness (nm)	RMS ^h^ (nm)	Standard Error of Roughness (nm)
1-for	2.26	0.73	11.81	0.26	39.88	159.59	101	1.64	32	1.72
1-rev	2.59	0.78	9.65	0.34	53.15	268.20				
2-for	4.14	0.84	11.90	0.42	33.15	322.82	150	1.77	19	1.20
2-rev	4.68	0.87	11.53	0.47	32.80	1026.60				
3-for	2.87	0.79	10.19	0.36	48.42	378.79	189	2.45	16	0.97
3-rev	3.51	0.83	10.05	0.42	45.60	1637.45				

Notes: ^a^ PCE: power conversion efficiency; ^b^ V_oc_: open-circuit voltage; ^c^ J_sc_: short-circuit photocurrent density; ^d^ FF: fill factor; ^e^ R_s_: series resistance; ^f^ R_sh_: shunt resistance; ^g^ Average thickness: the average thickness of TiO_2_ films; ^h^ RMS: roughness measurement of the surface of TiO_2_ films.
